# Supplementary material for: Testing the evolutionary drivers of malaria parasite rhythms and their consequences for host–parasite interactions
Source: Evol Appl. 2024 Jul 14;17(7):e13752. doi: 10.1111/eva.13752 (PMC11246599; doi:10.1111/eva.13752)
Supplement: Supplementary file 1 — Table S1. Table S2. Table S3. Table S4. [file EVA-17-e13752-s001.docx]

**Consequences of daily rhythms in host-parasite interactions during malaria infection – supplementary material**

Table S1 - Parasite density models. The three table sections show model selection for, respectively, Log(parasite density), Log(parasite peak density) or Cumulative parasite density as response variables, with Treatment as a fixed factor. Day PI (Day, as a fixed factor), Day x Treatment interaction, and Mouse ID (as random intercepts) were also included in the full parasite density model. Degrees of freedom (Df), log likelihood (Log lik), ΔAICc (the most parsimonious model is indicated with 0 in bold), and model weight (weight) are shown for each analysis. The coefficient (coef) and standard error of the mean (SE) for the three key questions are given for the most parsimonious model, with significant differences in bold. Specifically, the coefficients are as follows: Q1 = WT-LF treatment, using WT-DF as reference level; Q2 = WT-AL treatment, using WT-DF as reference level; Q3 = Per1/2-RF treatment, using Per1/2-AL as reference level.

| Model | Df | Log lik | ΔAICc | weight | Q1 coef ±SE | Q2 coef ±SE | Q3 coef ±SE |
| --- | --- | --- | --- | --- | --- | --- | --- |
| Log(Density) ~ Day + Treatment + (1 \| MouseID) | **21** | **-441.60** | **0** | **0.796** | **0.36 ± 0.15** | -0.14 ± 0.15 | 0.11 ± 0.16 |
| Log(Density) ~ Day + Treatment + Day *x* Treat + (1 \| MouseID) | 77 | -364.75 | 3.08 | 0.170 |  |  |  |
| Log(Density) ~ Day + (1 \| MouseID) | 17 | -449.27 | 6.36 | 0.033 |  |  |  |
| Log(Density) ~ Treatment + (1 \| MouseID) | 7 | -482.82 | 51.90 | 0 |  |  |  |
| Log(Density) ~ 1 + (1 \| MouseID) (null) | 3 | -490.13 | 58.24 | 0 |  |  |  |
| Log(Peak Height) ~ Treatment | **6** | **-1.27** | **0** | **0.991** | 0.23 ± 0.18 | **0.41 ± 0.18** | 0.11 ± 0.18 |
| Log(Peak Height) ~ 1 (null) | 2 | -12.01 | 9.36 | 0.009 |  |  |  |
| Cumul. Density ~ Treatment | **6** | **-496.73** | **0** | **0.927** | (6.5 ± 9.6) x 10^8^ | (10.7± 9.6)  x 10^8^ | (-1.6± 11.0)  x 10^8^ |
| Cumul. Density ~ 1 (null) | 2 | -478.94 | 5.07 | 0.073 |  |  |  |

Table S2 - Gametocyte density models. The four table sections show model selection for, Log(gametocyte density), Log(gametocyte peak density for the 1^st^ wave), Log(gametocyte peak density for the 2^nd^ wave), or Cumulative gametocyte density as response variables, with Treatment as a fixed factor. Day PI (Day, as a fixed factor), Day x Treatment interaction, and Mouse ID (as random intercepts) were also included in the full gametocyte density model. Degrees of freedom (Df), log likelihood (Log lik), ΔAICc (the most parsimonious model is indicated with 0 in bold), and model weight (weight) are shown for each analysis.. For the peak and cumulative density models, the coefficient (coef) and standard error of the mean (SE) for two of the key questions are given for the most parsimonious model. Specifically, the coefficients given are as follows: Q1 = WT-LF treatment, using WT-DF as reference level; Q2 = WT-AL treatment, using WT-DF as reference level.

| Model | Df | Log lik | ΔAICc | weight | | Q1 coef ±SE | Q2 coef ±SE |
| --- | --- | --- | --- | --- | --- | --- | --- |
| Log(Density) ~ Day + Treatment | **62** | **-418.66** | **0** | **1** | See Table S3 | |  |
| Log(Density) ~ Day + Treatment + Day *x* Treat | 20 | -494.59 | 34.64 | 0 |  | |  |
| Log(Density) ~ Day | 17 | -498.90 | 36.34 | 0 |  | |  |
| Log(Density) ~ Treatment | 6 | -596.94 | 208.4 | 0 |  | |  |
| Log(Density) ~ 1 (null) | 3 | -602.56 | 213.4 | 0 |  | |  |
| Log(1^st^ Peak Height) ~ Treatment | **5** | **-4.63** | **0** | **0.523** | 0.21 ± 0.22 | | -0.08 ± 0.22 |
| Log(1^st^ Peak Height) ~ 1 (null) | 2 | -9.51 | 0.18 | 0.477 |  | |  |
| Log(2^nd^ Peak Height) ~ 1 (null) | **2** | **-12.48** | **0** | **0.976** | NA | | NA |
| Log(2^nd^ Peak Height) ~ Treatment | 5 | -11.10 | 7.45 | 0.024 |  | |  |
| Cumul. Density ~ 1 (null) | **2** | **-315.83** | **0** | **0.927** | NA | | NA |
| Cumul. Density ~ Treatment | 5 | -314.72 | 8.38 | 0.015 |  | |  |

Table S3 - Gametocyte density dynamics from the most parsimonious model of parasite density, including a Day x Treatment interaction term. Coefficients and standard errors corresponding to two of the key questions; i.e. the WT-LF (Q1) and WT-AL (Q2) groups, relative to the reference group WT-DF. The intercept (Day 3 for WT-DF) is given.

| Day | Reference coef ±SE  (WT-DF) | Q1 coef ±SE | Q2 coef ±SE |
| --- | --- | --- | --- |
| 3 | 11.54 ± 0.61 (intercept) | 0.12 ± 0.87 | -0.82 ± 0.87 |
| 4 | 0.08 ± 0.83 | 2.41 ± 1.17 | 2.90 ± 1.17 |
| 5 | 3.96 ± 0.83 | 0.25 ± 1.17 | 0.47 ± 1.17 |
| 6 | 2.63 ± 0.83 | 1.73 ± 1.17 | 1.87 ± 1.17 |
| 7 | 2.03 ± 0.83 | 1.63 ± 1.17 | 1.42 ± 1.17 |
| 8 | 0.95 ± 0.83 | 0.37 ± 1.17 | 0.95 ± 1.17 |
| 9 | 0.36 ± 0.83 | 0.55 ± 1.17 | 0.69 ± 1.17 |
| 10 | 0.76 ± 0.83 | -0.81 ± 1.17 | 0.48 ± 1.17 |
| 11 | 2.41 ± 0.88 | -0.19 ± 1.21 | 3.33 ± 1.21 |
| 12 | 4.05 ± 0.88 | 0.89 ± 1.21 | 1.81 ± 1.21 |
| 13 | 5.27 ± 0.88 | -0.70 ± 1.21 | 0.18 ± 1.21 |
| 14 | 3.74 ± 0.88 | 0.03 ± 1.21 | 0.48 ± 1.21 |
| 15 | 4.11 ± 0.88 | -0.44 ± 1.21 | -1.07 ± 1.21 |
| 16 | 2.75 ± 0.88 | -0.58 ± 1.21 | -0.56 ± 1.21 |
| 17 | 1.73 ± 0.88 | -0.04 ± 1.21 | -0.91 ± 1.21 |

Table S4 Host virulence models. The two table sections show model selection for, Weight Loss or RBC loss as response variables, with Treatment as a fixed factorDegrees of freedom (Df), log likelihood (Log lik), ΔAICc (with most parsimonious model shown with 0 in bold), and model weight (weight) are shown. The coefficient (coef) and standard error of the mean (SE) for two of the key questions are given for the most parsimonious model, with significant differences in bold. Specifically, the coefficients given are as follows: Q1 = WT-LF treatment, using WT-DF as reference level; Q2 = WT-AL treatment, using WT-DF as reference level.

| Model | Df | Log lik | ΔAICc | weight | Q1 coef ±SE | Q2 coef ±SE | Q3 coef ±SE |
| --- | --- | --- | --- | --- | --- | --- | --- |
| Weight loss ~ Treatment | **6** | **-28.72** | **0** | **0.996** | 0.04 ± 0.54 | -1.3 ± 0.54 | 0.54 ± 0.54 |
| Weight loss ~ 1 (null) | 2 | -40.26 | 10.96 | 0.004 |  |  |  |
| RBC loss ~ 1 (null) | **2** | **-14.27** | **0** | **0.934** | NA | NA | NA |
| RBC loss ~ Treatment | 6 | -10.86 | 5.31 | 0.066 |  |  |  |
